# Supplementary material for: Impact of Parametrizations of the One-Body Reduced Density Matrix on the Energy Landscape
Source: J Phys Chem Lett. 2025 Apr 8;16(15):3822–31. doi: 10.1021/acs.jpclett.5c00308 (PMC12010429; doi:10.1021/acs.jpclett.5c00308)
Supplement: Supplementary file 1 — jz5c00308_si_001.pdf [file jz5c00308_si_001.pdf]

jz-2025-00308b.R1

Name: Peer Review Information for "Impact of Parametrizations of the One-Body Reduced Density Matrix on the Energy Landscape"

First Round of Reviewer Comments

Reviewer: 1

Comments to the Author

The manuscript « Impact of Parametrizations of the One-Body Reduced Density Matrix on the Energy Landscape » discuss the difficulties of minimizing the energy, functional of the 1-RDM, wrt. the 1RDM in its spectral representation. This topic is timely and the optimization problem in RDMFT is usually not addressed enough. This manuscript aims to highlight potential problems when expressing the 1-RDM in terms of its eigenvectors and eigenvalues, which is what is done in the literature as all (but Hartree-Fock) functionals for quantum chemistry are expressed in this spectral representation. The analysis is performed on several parametrizations for the orthonormal transformation. The manuscript is clear and well written, with enough details in the appendices to follow and reproduce the work. In my opinion, the manuscript can be published in JPCL after minor modifications :

1) TOC : in the TOC, there are figures that are not in the manuscript. One using the exponential and the other the Cayley transformation. I think those additional figures (maybe same for the Householder transformation and Given's rotations) could be added to the appendices as well.

2) « Nevertheless, considering the difficulty to design a robust and efficient algorithm to optimize the energy in RMDFT, we will briefly introduce RDMFT which also allows us to introduce our notation. ». I don't really understand the sentence, I don't see the connection between introducing RDMFT and your notation, and the fact that it is difficult to design a robust and efficient algorithm. This sentence should be reformulated.

3) Figure 1, please provide the expressions for the Muller functional in the 1-RDM gamma representation and the [x_12, n_1] representation. Indeed, I only know the Muller functional in the spectral representation, I didn't know there was one depending directly on the 1RDM gamma.

4) « iteratively diagonals a generalized Fock matrix » → diagonalizes

5) « allows us the to write » → allows us to write

6) « in particular not block the convergence, even if they are minima », I don't get this footnote formulation, needs rewriting ?

7) Eq. 32 : please don't put references inside the equations.

Reviewer: 2

Comments to the Author

Review for Impact of parametrizations of the one-body reduced density

matrix on the energy landscape

1. What is the major advance reported in the paper?

This article provides an analysis of the parametrization of the one-body

reduced density matrix, especially comparing the critical points of the

energy as:

1- a function of the density matrix, 2- a function of occupation numbers

and orbitals.
The energy is written as a composition of a map from occupation numbers and orbitals to density matrix and the map from density matrix to energy: \tilde E(n,x) = E(\gamma(n,x)), for which is the gradient is simply \nabla E_{n,x} = (\nabla \gamma(n,x))^T \nabla E_{\gamma}.

The authors study when this quantity is zero (critical point) and in particular are interested in spurious critical points.
For this they compute the determinant of the matrix (\nabla \gamma(n,x))^T and characterize when it is zero for several parametrizations of the occupation numbers and show that for problematic parametrizations, and for degenerate occupation numbers, the spurius points can only be saddle points of the energy.

They conclude that the parametrization "introduces additional critical points for degenerate natural occupation numbers."
Unfortunately one argument is missing here. To have a critical point, \nabla E_{\gamma} must be in the kernel of the matrix (\nabla \gamma(n,x))^T. But this is not checked, as it is only studied when the matrix \nabla \gamma(n,x) is singular.
Hence, the study seems incomplete, and a key phrase of the abstract as well as the conclusion seems incorrectly stated.

2. What is the immediate significance of this advance?

This is not so clear. The practical impact of these findings seems to be that second-order methods need to be used in the optimization because they are able to escape saddle points. But no numerical evidence is given regarding this.

Maybe the message would be that the optimization methods have to be expressed in terms of the density matrix as the energy landscape has better properties, and then translated to the orbital and occupation numbers ? (in this direction, see e.g. this paper: Eric Cancès, Gaspard Kemlin, and Antoine Levitt. "Convergence analysis of direct minimization and self-consistent iterations." SIAM Journal on Matrix Analysis and Applications 42.1 (2021): 243-274.) This is shortly mentioned in the conclusion.

3. Technical suggestions

Formula (6) seems correct but its derivation could highly be simplified using the chain rule. In general the proofs seem quite involved and could probably be simplified.

Other comments:

- p.2, l.12-17 I do not understand this phrase
- p.2, l.34 What is an increase in the nonlinearity ?
- p.3, l.6 gradient should be jacobian
- p.6, l.46 "the hessian can only positive semi-definite if and only if E_ij = 0" - there is one extra only.

- p.7, (24) there is an error in the matrices, although the final result

seems correct. (AB)^T = B^T A^T

Reviewer: 3

Comments to the Author

In this manuscript the authors analyze the total energy minimization in methods based on the one-body density matrix (1RDM), notably Reduced Density Matrix Functional Theory (RDMFT).

Usually, the energy minimization is not performed with respect to the 1RDM itself but rather with respect to its eigenvectors and eigenvalues, i.e., the natural orbitals (NO) and the occupation numbers, respectively. This is because most 1RDM energy functional approximations are formulated in terms of NOs and occupation numbers. However, this approach results in the loss of convexity of the energy functional. The manuscript therefore analyzes the presence of additional critical points (i.e., the points besides the proper ones, in which the gradient of the energy functional is zero due to the gradient of the 1RDM in parameter space being zero). The authors conclude that the important additional critical points are the ones occurring at degenerate natural occupation numbers. They find, however, that these critical points can only be saddle points, and thus can be identified using a second-order algorithm.

I find this manuscript very interesting, clearly written, and self-contained. All the derivations of the main equations are provided in the appendices, ensuring that the flow of the paper is not cut while still offering full details for interested readers.

The conclusions of this work are highly significant and point to the fact that one should favor energy functionals of the 1RDM. One point emphasized by the authors is the extension of this analysis to complex orbitals. This is particularly important since RDMFT is also used in solids, and it would be interesting to see whether and how additional complexities arise. In this regard, it is a pity that the authors do not cite all relevant works on RDMFT in solids. I suggest that they include some references. For the rest, I have only a few minor corrections/typos that I will list below.

For all above, I believe that this manuscript meets the criteria for publication in The Journal of Physical Chemistry Letters.

Corrections/typos:

1) page 2, lines 1-4, first column: The sentence "As in Hartree–Fock theory, the 1RDM should even be idempotent ni ∈ {0, 1},

a restriction which also often imposed on the Kohn–Sham system in DFT." sounds strange. The 1RDM is generally not idempotent, except in independent particle methods such HF or DFT. The authors should consider reformulating this sentence for clarity.

2) In Equation 2 the laplacian is denoted as \Delta, but this notation is not specified in the main text. It would be helpful to clarify it.

3) page 4, Lines 21-23, first column: the sentence "The first step is thus to determine when is ∇n,xγ singular,

that is, when do we have det (∇n,xγ) = 0." sounds strange, as it appears to be phrased as a question without being one.

4) Lines 5 , second column: "a saddle points"—>"a saddle point"

Author's Response to Peer Review Comments:

Answer to Reviewers

Reviewer 1:
Comments:

The manuscript « Impact of Parametrizations of the One-Body Reduced Density Matrix on the Energy Landscape » discuss the difficulties of minimizing the energy, functional of the 1-RDM, wrt. the 1RDM in its spectral representation. This topic is timely and the optimization problem in RDMFT is usually not addressed enough. This manuscript aims to highlight potential problems when expressing the 1-RDM in terms of its eigenvectors and eigenvalues, which is what is done in the literature as all (but Hartree-Fock) functionals for quantum chemistry are expressed in this spectral representation. The analysis is performed on several parametrizations for the orthonormal transformation. The manuscript is clear and well written, with enough details in the appendices to follow and reproduce the work. In my opinion, the manuscript can be published in JPCL after minor modifications :

1) TOC : in the TOC, there are figures that are not in the manuscript. One using the exponential and the other the Cayley transformation. I think those additional figures

(maybe same for the Householder transformation and Given's rotations) could be added to the appendices as well.

In the case presented (specificity of 2 orbitals) Givens and exponential as well as Householder and Cayley give the same parametrisation (up to a factor) so we presented Cayley and exponential in the TOC. The exponential was already in Fig. 1, but we have added Cayley as well for completeness.

2) « Nevertheless, considering the difficulty to design a robust and efficient algorithm to optimize the energy in RMDFT, we will briefly introduce RDMFT which also allows us to introduce our notation. ». I don't really understand the sentence, I don't see the connection between introducing RDMFT and your notation, and the fact that it is difficult to design a robust and efficient algorithm. This sentence should be reformulated. The phrasing was indeed confusing and we have changed this sentence.

3) Figure 1, please provide the expressions for the Muller functional in the 1-RDM gamma representation and the [x_12, n_1] representation. Indeed, I only know the Muller functional in the spectral representation, I didn't know there was one depending directly on the 1RDM gamma

We have added the expression of the Müller functional (as functional of the 1-RDM) in equation (3).

4) « iteratively diagonals a generalized Fock matrix » → diagonalizes

5) « allows us the to write » → allows us to write These mistakes have been corrected.

6) « in particular not block the convergence, even if they are minima », I don't get this footnote formulation, needs rewriting ?

What we intended here, is to emphasis that the update of $C^{(n)}$ prevent the minimization form being stuck at critical points due to the orbital parametrisation, even if these points are local minima. We have rephrased the footnote so that it is clearer.

7) Eq. 32 : please don't put references inside the equations.

We have added a sentence before equation 34 (formerly 32) and placed the reference there.

Reviewer: 2

Recommendation: This paper may be publishable, but major revision is needed; I would like to be invited to review any future revision.

Comments:

Review for Impact of parametrizations of the one-body reduced density matrix on the energy landscape

1. What is the major advance reported in the paper?

This article provides an analysis of the parametrization of the one-body reduced density matrix, especially comparing the critical points of the energy as:

1- a function of the density matrix, 2- a function of occupation numbers and orbitals.

The energy is written as a composition of a map from occupation numbers and orbitals to density matrix and the map from density matrix to energy: \tilde E(n,x) = E(\gamma(n,x)), for which is the gradient is simply \nabla E_{n,x} = (\nabla \gamma(n,x))^T \nabla E_{\gamma}.

The authors study when this quantity is zero (critical point) and in particular are interested in spurious critical points. For this they compute the determinant of the matrix (\nabla \gamma(n,x))^T and characterize when it is zero for several parametrizations of the occupation numbers and show that for problematic parametrizations, and for degenerate occupation numbers, the spurius points can only be saddle points of the energy.

They conclude that the parametrization "introduces additional critical points for degenerate natural occupation numbers."

Unfortunately one argument is missing here. To have a critical point,

\nabla E_{\gamma} must be in the kernel of the matrix (\nabla \gamma(n,x))^T. But this is not checked, as it is only studied when the matrix \nabla \gamma(n,x) is singular.

Hence, the study seems incomplete, and a key phrase of the abstract as well as the conclusion seems incorrectly stated.

The statement was indeed not complete and we rephrased it all along the paper, to indicate that such spurious points can appear but do not necessarily have to. It is unfortunately not possible to provide a better characterisation of when do these spurious points happen as is depends on the values taken by \nabla_{\gamma} E, which depends on the functional and the system under study. Numerically the null space will be very large. So we believe that this problem does occur in practice as often observe slow convergence of the SCF procedure in RDMFT, that is drastically improved by using second-order methods or optimising directly w.r.t. the 1-RDM. The conclusion has been rephrased to emphasise this point. Moreover, to show that spurious

critical points due to the occupation degeneracy do exist, we have added an explicit example in Appendix F.

2. What is the immediate significance of this advance?

This is not so clear. The practical impact of these findings seems to be

that second-order methods need to be used in the optimization because they are able to escape saddle points. But no numerical evidence is given regarding this. Maybe the message would be that the optimization methods have to be expressed in terms of the density matrix as the energy landscape has better properties, and then translated to the orbital and occupation numbers ? (in this direction, see e.g. this paper: Eric Cancès, Gaspard Kemlin, and Antoine Levitt. "Convergence analysis of direct minimization and self-consistent iterations." SIAM Journal on Matrix Analysis and Applications 42.1 (2021): 243-274.) This is shortly mentioned in the conclusion.

In this paper we point out the problems that may occur by optimizing w.r.t. parameters instead of the 1-RDM (and rule out some of them). A possible solution we indeed mention at the end is to do the optimization w.r.t. the 1-RDM itself, some works have already tried this, with encouraging results and have been added as references to the conclusion. We also think that a second order method can suffice to reach a good convergence, this is also mentioned in the paper and even if we do not provide numerical evidences in this letter (the latter is intended to be analytical derivations and not numerical results), we point out to references available in the literature. We precise that we do not favour one approach over the other (i.e. second order method or optimisation w.r.t. the 1-RDM) as our findings do not allow us to do so. The aim of the paper is not to formulate a new algorithm, but to provide an explanation for the behaviour of several energy optimization algorithms.

3. Technical suggestions

Formula (6) seems correct but its derivation could highly be simplified using the chain rule. In general the proofs seem quite involved and could probably be simplified.

We do agree that simpler proofs would be desirable, unfortunately we have to take into account the fact that the 1-RDM is an MxM matrix while we have M(M+1) variables making some derivations more involved. The fact that the X parameter in the Cayley and

exponential parametrizations, has to be regarded as a matrix or a vector depending on the context also make it harder to write down simple derivations.

In the case of equation 8 (former 6), we are not sure about the reviewers has in mind. Although this is probably not the one he refers to, we point out that the straightforward chain rule $\frac{\partial \gamma_{pq}}{\partial x_{ij}} = \sum_k \frac{\partial U_{pk}}{\partial x_{ij}} n_k U^T_{kq} + \sum_l U_{pl} n_l \frac{\partial U^T_{lq}}{\partial x_{ij}}$ does not give the simple compact expression we hope for anymore, since we cannot use $\frac{\partial U^T}{\partial x} = U \frac{\partial U^T}{\partial x}$ anymore (as we cannot decouple the occupations and orbitals that nicely and thus do not have a simple matrix product).

Other comments:

- p.2, l.12-17 I do not understand this phrase
- p.2, l.34 What is an increase in the nonlinearity ?

We have rephrased these parts for clarity.

- p.3, l.6 gradient should be jacobian

$\nabla_{n,x}$ is a gradient and $\nabla_{n,x}\gamma$ a Jacobian, we have added this precision for clarity

- p.6, l.46 "the hessian can only positive semi-definite if and only ifE_ij = 0" - there is one extra only.

The first 'only' has been removed.

- p.7, (24) there is an error in the matrices, although the final resultseems correct. (AB)^T = B^T A^T

Note that we do not have a matrix product in equation 23 (former 21) but something akin to a Kronecker product, so the order of the transposed matrix does not matter in equation 26 (former 24).

Reviewer: 3

Recommendation: This paper is publishable subject to minor revisions noted. Further review is not needed.

Comments:

In this manuscript the authors analyze the total energy minimization in methods based on the one-body density matrix (1RDM), notably Reduced Density Matrix Functional Theory (RDMFT).
Usually, the energy minimization is not performed with respect to the 1RDM itself but rather with respect to its eigenvectors and eigenvalues, i.e., the natural orbitals (NO) and the occupation numbers, respectively. This is because most 1RDM energy functional approximations are formulated in terms of NOs and occupation numbers. However, this approach results in the loss of convexity of the energy functional. The manuscript therefore analyzes the presence of additional critical points (i.e., the points besides the proper ones, in which the gradient of the energy functional is zero due to the gradient of the 1RDM in parameter space being zero). The authors conclude that the important additional critical points are the ones occurring at degenerate natural occupation numbers. They find, however, that these critical points can only be saddle points, and thus can be identified using a second-order algorithm.
I find this manuscript very interesting, clearly written, and self-contained. All the derivations of the main equations are provided in the appendices, ensuring that the flow of the paper is not cut while still offering full details for interested readers.
The conclusions of this work are highly significant and point to the fact that one should favor energy functionals of the 1RDM. One point emphasized by the authors is the extension of this analysis to complex orbitals. This is particularly important since RDMFT is also used in solids, and it would be interesting to see whether and how additional complexities arise. In this regard, it is a pity that the authors do not cite all relevant works on RDMFT in solids. I suggest that they include some references. For the rest, I have only a few minor corrections/typos that I will list below.
For all above, I believe that this manuscript meets the criteria for publication in The Journal of Physical Chemistry Letters.

Corrections/typos:

1) page 2, lines 1-4, first column: The sentence “As in Hartree–Fock theory, the 1RDM should even be idempotent ni ∈ {0, 1}, a restriction which also often imposed on the Kohn–Sham system in DFT.” sounds strange. The 1RDM is generally not idempotent,

except in independent particle methods such HF or DFT. The authors should consider reformulating this sentence for clarity.
The sentence has been slightly modified to make it clear that we are talking about HF and DFT (as correctly stated by the reviewer).

2) In Equation 2 the laplacian is denoted as \Delta, but this notation is not specified in the main text. It would be helpful to clarify it.

We have specified the meaning of Δ_r in the main text.

3) page 4, Lines 21-23, first column: the sentence “The first step is thus to determine when is ∇n,xγ singular, that is, when do we have det (∇n,xγ) = 0.” Sounds strange, as it appears to be phrased as a question without being one.
The sentence has been rephrased to be affirmative.

4) Lines 5 , second column: “a saddle points”—>”a saddle point” The typography mistake has been corrected.

jz-2025-00308b.R2

Name: Peer Review Information for "Impact of Parametrizations of the One-Body Reduced Density Matrix on the Energy Landscape"

Second Round of Reviewer Comments

Reviewer: 2

Comments to the Author

the authors have taken into account my comments, and I believe that the paper may now be published.

Author's Response to Peer Review Comments:

After verification, it appears that the Similarity Index of 67% is by comparing our submitted paper with its preprint version on arXiv. As per ACS policy on preprint, we therefore believe

that no modification is required provided that the mention "This document is the unedited Author's version of a Submitted Work that was subsequently accepted for publication in JPC Letter, copyright © [include copyright notice from the published article] after peer review. To access the final edited and published work see [insert ACS Articles link]" is added to the arXiv after publication.
